# Biochemical and Molecular Aspects of Phosphorus Limitation in Diatoms and Their Relationship with Biomolecule Accumulation

**DOI:** 10.3390/biology10070565

**Published:** 2021-06-22

**Authors:** José Pablo Lovio-Fragoso, Damaristelma de Jesús-Campos, José Antonio López-Elías, Luis Ángel Medina-Juárez, Diana Fimbres-Olivarría, Corina Hayano-Kanashiro

**Affiliations:** Departamento de Investigaciones Científicas y Tecnológicas, Universidad de Sonora, Blvd. Colosio y Sahuaripa, Colonia Centro, Hermosillo, Sonora CP 83000, Mexico; pablo.lovio@gmail.com (J.P.L.-F.); ddjesus.campos@gmail.com (D.d.J.-C.); antonio.lopez@unison.mx (J.A.L.-E.); luis.medina@unison.mx (L.Á.M.-J.); diana.fimbres@unison.mx (D.F.-O.)

**Keywords:** diatoms, phosphorus, biomolecules, lipid accumulation, molecular mechanisms

## Abstract

**Simple Summary:**

Phosphorus (P) is a key nutrient involved in the transfer of energy and the synthesis of several cellular components. It has been reported that P limitation in diatoms induces the synthesis of biomolecules and the accumulation of storage compounds, such as pigments, carbohydrates and lipids, with diverse biological activities, which can be used in diverse biotechnological applications. However, the molecular and biochemical mechanisms related to how diatoms cope with P deficiency are not clear, and research into this has been limited to a few species. The integration of results obtained from omics sciences could provide a broad understanding of the response of diatoms to P limitation, and the information obtained could help to solve challenges such as biomass production, by-products yield and genetic improvement of strains.

**Abstract:**

Diatoms are the most abundant group of phytoplankton, and their success lies in their significant adaptation ability to stress conditions, such as nutrient limitation. Phosphorus (P) is a key nutrient involved in the transfer of energy and the synthesis of several cellular components. Molecular and biochemical mechanisms related to how diatoms cope with P deficiency are not clear, and research into this has been limited to a few species. Among the molecular responses that have been reported in diatoms cultured under P deficient conditions is the upregulation of genes encoding enzymes related to the transport, assimilation, remobilization and recycling of this nutrient. Regarding biochemical responses, due to the reduction of the requirements for carbon structures for the synthesis of proteins and phospholipids, more CO_2_ is fixed than is consumed by the Calvin cycle. To deal with this excess, diatoms redirect the carbon flow toward the synthesis of storage compounds such as triacylglycerides and carbohydrates, which are excreted as extracellular polymeric substances. This review aimed to gather all current knowledge regarding the biochemical and molecular mechanisms of diatoms related to managing P deficiency in order to provide a wider insight into and understanding of their responses, as well as the metabolic pathways affected by the limitation of this nutrient.

## 1. Introduction

Diatoms are a diverse and heterogeneous group of microalgae and are considered the most abundant group of phytoplankton distributed around the world. They contribute approximately 20 to 40% of global primary production, and they appear to have a significant adaptation ability to stress conditions [1,2]. Diatoms, like all other microalgae species, have specific requirements for their growth and survival, including both biotic and abiotic factors, such as light, temperature, available carbon dioxide (CO_2_) concentration, pH, salinity and the contribution of macronutrients such as nitrogen (N), phosphorus (P) and silicon (Si, which is essential for diatoms) and trace elements such as metals and vitamins [3,4,5,6]. It has been reported that nutrient availability in aquatic environments varies depending on the season, the region and anthropogenic factors [7]. Generally, however, the concentration of P in aquatic environments tends to be low [8,9]; given this, microalgae have developed strategies to cope with P limitation.

Microalgae under P-limited conditions can quickly incorporate phosphates and store them as high molecular weight compounds called polyphosphates (PolyP); this complex process is known as luxury P uptake [10,11]. Under favorable P concentrations, microalgal cells contain 1% (*w*/*w*) of P in dry weight (dw), and luxury uptake increases the P content up to 4–6% (dw) [12,13,14,15,16,17]. The P obtained during this process is stored in vacuoles as PolyP, and these molecules are metabolized when P is scarce [10,11]. Moreover, physiological studies have demonstrated that the diatom’s ability to capture P depends on whether they are benthic or planktonic and is also species-dependent [4,18]. Benthic diatoms can forage the bottom to compensate for the lack of this nutrient through chemokinetic and chemotactic responses, as has been observed in *Seminavis robusta*, while planktonic species can move throughout the water column searching for the resource [18]. According to Lin et al. [4], microalgae require coordinated cellular feedback systems to detect the P concentration inside and outside the cell. Once the P reaches the cell surface it is transported through the cell membrane by binding with transmembrane proteins; this process consumes notable quantities of energy, where ATP is involved.

The role of P inside the cell includes several functions; this element is present in the phospholipids of the cellular membrane as one of the main constituents. It is also a fundamental component of nucleic acids (DNA and RNA) and plays a key role in the production of chemical energy in the form of ATP and NADPH, which are indispensable molecules in the photosynthesis process. P is an important element for the synthesis of proteins via ribosomal RNA and helps to regulate their function [4,19,20]. Diatoms can use different P sources; the inorganic form (Pi, PO_4_^3−^) is the most used due to its rapid and easy assimilation. Despite the above, when Pi is scarce, dissolved organic P (DOP) in the form of polyphosphates, phosphoesters and phosphonates can be used through conversion to Pi, but at a high energetic cost due to the enzymatic activity of the phosphodiesterases and phosphatases [4,19,20].

When nutrients are scarce, microalgal cells need to redirect their carbon metabolism in order to survive. It has been observed that under P limitation, biomolecule production increases during the transition from the logarithmic to stationary phase in the marine diatoms *Cylindrotheca closterium*, *Thalassiosira pseudonana* and *Skeletonema costatum* [19,21]. Physiologically, microalgae tend to synthesize carbohydrates and proteins, which are fundamental constituents in vital processes during the logarithmic phase; however, carbohydrates tend to be augmented with the culture age [22,23]. From the biochemical point of view, this response could be due to the reduction of the requirements for carbon structures; consequently, the excess of carbon is redirected to the synthesis of storage compounds such as triacylglycerides (TAGs) or is excreted as extracellular polymeric substances (EPSs), which are used in several biotechnological applications [19,24].

The P limitation effects in microalgae have been little studied in comparison with other nutrients such as nitrogen; particularly in diatoms, most studies have been focused on biochemical responses. On the other hand, the emergence of omics sciences has allowed the development of a broader understanding of the responses of microalgae to P deficiency, although in diatoms the knowledge that has been generated has been limited to only a few species [25,26]. Due to the importance of P as a key nutrient involved in the transfer of energy and synthesis of several cellular components, it is of interest to study its effect on the biochemical, molecular and physiological response of diatoms when Pi is limited. In this review, we aimed to generate a deeper knowledge than has already been generated on diatoms and their physiological, biochemical and molecular responses to P limitation. We found it interesting that similar responses occur among different species, although there are some other responses that differ among them. Moreover, we gathered recent information that, to our knowledge, has not been considered in other reviews related to this topic.

## 2. P Effect on Growth and Biomass Production

Nutrient availability determines species distribution [27]. When nutrients are scarce, cells tend to decrease their proliferation and redirect their energy to survive under stress conditions. Diatoms cultured under P deficiency have been reported to be more susceptible to the P concentration than some flagellated microalgae species [1,27,28].

Finenko and Krupatkina-Akinina [29] evaluated the effect of different Pi concentrations on the growth rates of seven diatoms cultured in Goldberg’s medium modified by Kabanova [30], finding that as the Pi concentration increased in the medium, the cell division rate also increased. They observed that when diatoms were cultured in a Pi-free medium, cell division was possible due the Pi reservoirs that these species may have had before the experiment, until eventually cell division stopped; they also observed that organisms which were acclimated in Pi-free medium responded faster to Pi supply than those that has been previously cultured under any Pi concentration. Despite the above, several studies have suggested that variations in the cell division rate and biomass can vary among diatom species in response to P availability. *P. tricornutum* stopped its cell division after two days of being exposed to an absence of P; *T. pseudonana* continued their cell division for five days under similar conditions [1,31,32]. Brembu et al. [19] pointed out that, assuming that both diatoms were cultured under the same conditions, this is probably because *P. tricornutum* is more sensitive than *T. pseudonana* to P limitation.

Concerning total biomass content, a decrease in the microalgal biomass is a typical response to low P concentrations in culture media; in contrast, limitation or starvation of other nutrients, such as N, have been shown to have unfavorable effects on the growth of many species of microalgae, including diatoms. However, this response tends to be species-dependent. In contrast to P limitation, P excess has rarely been studied. Yongmanitchai and Ward [33] cultured *P. tricornutum* in Mann Myers medium and increased the P concentration from 2.5-fold to 5-fold higher than the control, and they found no significant differences in total biomass content among all the treatments. Recently, Lopes et al. [34] cultured *P. tricornutum* in F/2 medium and increased the P concentration from 1.5-fold to 3-fold higher than the control, finding the same results as Yongmanitchai and Ward [33]. On the other hand, in studies carried out in *Chaetoceros muelleri* grown in F medium, P concentration was doubled compared to the control, and a higher content of dry biomass was found in the control treatment compared to the P excess treatment. However, no significant differences were found in cell density between the control and excess concentrations [35].

Another response to nutrient limitation (such as N and P) and other abiotic factors, such as light, is morphological changes, such as the cellular volume [5,36,37]. Under P starvation in diatoms, Liu et al. [38] found that the cell size of *Thalassiosira weissflogii* was larger (up to 1.48 and 2.67 times) than those kept in favorable P concentrations. This effect of P concentration on cell volume was also observed in *Cyclotella nana* [39]. Lippemeier et al. [40] suggested that there is an inverse relationship of cell volume with cell division rates, allowing the accumulation of storage biomolecules.

## 3. P Sensing in Different Organisms

All living organisms have different mechanisms to sense the presence and availability of metabolites and nutrients required to maintain cell homeostasis and, therefore, their proper functioning and survival. In this section will be discussed what is known about P sensing mechanism in diatoms compared with those in other organisms. The P sensing system is not clear in diatoms; however, it is thought that it is defined by the species, strains and also by the exogenous P supply, as well as its reserve within the cells [13]. Therefore, knowledge of the P sensing mechanisms in different organisms, such as prokaryotes (bacteria) and eukaryotes (yeasts and higher plants), could help to elucidate these mechanisms in microalgae in general, as well as in diatoms.

In bacteria, the responses to P deficiency are controlled by a sensing system, consisting of two components (PhoR and PhoB), that results in the transcription of a set of genes necessary to respond to P deficiency. This system is known as Pho regulon, whose components are the PhoR and PhoB proteins [41]. PhoR is a transmembrane protein located in the cell membrane, which perceives the presence of Pi on the cell surface or in the cytosol. Pi dissociates from PhoR to subsequently phosphorylate PhoB in the cytoplasm, allowing it to bind to specific regulatory regions of DNA located upstream of the Pho regulon genes, known as Pho boxes; this union allows the initiation of transcription of the genes involved in the synthesis of intracellular Pi transport proteins and, in many cases, those that are involved in the use of dissolved organic P [4,42]. In cyanobacteria (also called “blue-green algae”), there is evidence that some Pho regulon genes are induced before others; this has been attributed to the fact that there are probably components involved in the stress response of P that are regulated by a different signaling cascade, or by changes in the mechanism of PhoR–PhoB that allows a certain response [43].

In yeast, the Pi-response signaling pathway is called the PHO pathway [44]. This pathway is regulated mainly by the transcription factor (TF) Pho84 (which is a transceptor protein, which can detect and transport extracellular Pi); Pho4; Pho80–Pho85, a cyclin-dependent kinase (CDK) complex; and Pho81, a cyclin-dependent kinase (CDK) inhibitor [44,45]. When cells are under P limitation, the CDK inhibitor Pho81 inactivates Pho80–Pho85 by changing its conformation preventing Pho4 from being phosphorylated; then, Pho4 interacts with the TF Pho2 to be imported into the nucleus, to activate the genes belonging to the Pho regulon [44,46,47]. The yeast Pho regulon is composed of genes encoding high-affinity Pi transporters (Pho84, Pho89) and acid phosphatases (Pho5, Pho11, Pho12) [48]. The Pi transport system in yeast consists of two types of Pi transporters: high-affinity transporters (Pho84, Pho89) located in the plasma membrane, and low-affinity transporters (Pho87, Pho90) also located in the plasma membrane and in the vacuolar membrane (Pho91) [49].

In higher plants, changes in Pi levels in the soil are seen at the tips of the roots, mainly in the root apical meristem (RAM) of the primary roots [50]. Studies in Arabidopsis have reported that the main genes involved in external Pi sensing, development and remodeling of roots are low phosphate root (*LPR1*, *LPR2*), phosphate deficiency response 2 (*PDR2*), aluminum activated malate transporter 1 (*ALMT1*) and sensitive to proton rhizotoxicity 1 (*STOP1*) [50,51,52].

A transcriptomic study in Arabidopsis revealed that 70% of the genes involved in the response to P availability in the plant are directly related to the external concentration of this nutrient in the environment, while the remaining genes are systemically regulated by the internal Pi status. From this 30%, the majority of the genes are regulated by the TF Phosphate Starvation Response 1 (PHR1), and they are responsible for Pi acquisition, allocation, recycling and homeostasis [53]. Another TF involved in the response to Pi starvation is WRKY6, which is involved in the regulation of the expression of the gene *PHOSPHATE1* (*PHO1*). *PHO1* codifies a membrane protein which is responsible for transporting Pi from root epidermal and cortical cells to the xylem [54,55].

Despite an increase in transcriptomic studies focused on the response to P limitation in diatoms, the mechanisms by which diatoms can sense the presence or absence of nutrients have recently begun to be described. Transcriptomic and enzymes characterization studies in diatoms have found that several alkaline phosphatases (APases) belong to the phoA and phoD gene families [56,57,58]. APases hydrolyze DOP present in the environment when Pi is no longer available in order to be easily assimilated by diatoms [59]. On the other hand, P transporter proteins found in diatoms show homology with members of the SLC34 and SLC20 gene families, which are distributed in all organisms [1,60,61,62]. The results obtained so far have demonstrated that the upregulation of genes encoding P transport proteins and APases is one of the main strategies that diatoms use to cope with P limitation [1,31,56,57,60,61].

As can be seen, prokaryote and eukaryote organisms studied so far have complex and sophisticated mechanisms to sense and properly use Pi, to guarantee the correct functioning of all biological processes. As mentioned before, in diatoms, there is not enough information about P sensing; however, recent studies have been identified P transporters and APases that are homologs to phoA, phoD, SLC34 and SLC20 [1,31,32,56,57,58,60,61], which are known gene families involved in P responses in different organisms such as bacteria and yeasts. It is suggested that even in these organisms that have already been studied, it is still necessary to fully elucidate how they respond to the availability of this nutrient [63]. Although, the current information about P limitation in diatoms has increased and are some clues that the P mechanisms could be similar in diatoms and other organisms, more studies are needed to fully comprehend the diatoms responses to the availability of P.

## 4. Molecular Responses Directly Related to P in Diatoms

### 4.1. P Uptake, Assimilation and Recycling Mechanisms of Diatoms under P Limitation

Transcriptomic analyses in diatoms have allowed us to identify the expression pattern of genes under P stress involved in several metabolic pathways, supporting a global understanding of the response of these organisms. When marine phytoplankton face a deficiency of Pi, one common strategy is the induction of high-affinity transporters of phosphates, APases and vacuolar transporter proteins as a response to fulfill cellular requirements and ensure their survival until environmental conditions become favorable again (Table 1).

Lin et al. [58] identified and characterized an extracellular alkaline phosphatase (PtAPase) from *P. tricornutum*. They observed that the gene encoding for PtAPase is highly sensitive to P concentration in the medium, finding a high induction from day 2 to day 4 of the start of the culture under low P concentrations. Then, under low P concentrations, PtAPase activity was measured, with the finding that it remained stable from day 4 to day 7, although the expression of the gene that encodes for that enzyme was almost downregulated. Conversely, at higher P concentrations, no activity of this enzyme was detected. The alignment analysis of the amino acid sequence of this PtAPase suggested that it belongs to the PhoA family, and it has also been reported that its activity is favored by divalent metal ions such as Mn^+2^, Mg^+2^ and Ca^+2^ at a 5 mM concentration. According to the authors, this alkaline phosphatase was the first to be purified in diatoms.

There are reports that some organisms use phosphite, hypophosphite and phosphine as alternative sources of P [64,65]. Studies by Fu et al. [64] evaluated the gene expression of a high-affinity transporter of *T. pseudonana*, *TpPHO*, cultured under different concentrations of phosphine, as an alternative source of P, as well as the activity of alkaline phosphatase. A similar cell density was observed at all phosphine concentrations, reaching the stationary phase around day 7 of the culture. The cell mortality rate was higher in the treatment with a higher concentration of phosphine compared to the other treatments with lower concentrations. Fu et al. [64] observed that when *T. pseudonana* is cultured under a 4 µM phosphate concentration and 0.022 µM phosphine, the induction of *TpPHO* is higher compared to control and high phosphine treatments, suggesting that phosphine induces the transcription of this gene, and therefore the assimilation of phosphates. On the other hand, it was detected that at high phosphine concentrations, the alkaline phosphatase activity was negatively affected. Based on these results, the authors suggested that phosphine has a dual effect on *T. pseudonana*, stimulating its growth under P-limiting conditions and low concentrations of phosphine, while high concentrations of phosphine inhibit the vitality of cells.

Under P deficiency, it has been observed that the genes encoding for the synthesis of ribosomal proteins are repressed and inhibit protein translation to conserve P in the rRNA. The central function of RNA is protein synthesis, involving mRNAs, rRNAs and tRNAs, and it is also believed to be involved in the regulation of gene expression. The P present in the RNA molecule is frequently considered the largest source of non-storage P in photosynthetic organisms, and any attempt to increase the efficient use of P in the plant growth and algae should consider economizing the use of RNA [66]. P is a biologically important element that is involved in the synthesis of P-rich ribosomal RNA when microalgae are exponentially growing [1,67]. However, this response may be nonspecific for nutrient limitation, as it may be due to decreased growth rates of any stress-generating factor [1]. On the other hand, among the genes that show high induction under P-deficient conditions in *T. pseudonana*, one that encodes a putative PUF (from Pumilio and FBF) family protein was found. PUF proteins are characterized by binding to the 3′UTR regions to repress gene expression, affecting mRNA translation and stability; although they have various functions, PUF proteins have not been characterized in any species of marine eukaryotic phytoplankton. Moreover, the differential expression of this gene has not been detected in limitation of N, Fe or Si in *T. pseudonana*, suggesting that this response may be specific to P limitation [1,68,69]. These findings are interesting because, after further and deeper studies, PUF proteins could be considered a marker of P deficiency in microalgae.

### 4.2. Regulatory Elements of Gene Expression under P Limitation in Diatoms

Transcriptomic studies have made it possible to identify the gene expression pattern of some diatoms upon P limitation, allowing a broad perspective of the metabolic pathways that could be induced or repressed to cope with this condition. However, it has been observed at the molecular level that these mechanisms are highly complex, since there are elements that regulate gene expression at the transcriptional, posttranscriptional, translational and posttranslational levels [70]. Regulatory elements of gene expression are those that control when, where and the levels of production of specific gene products (RNA or proteins) in response to genetic or environmental stimuli [71,72]; this has been corroborated in several studies, from bacteria to plants and mammals [73,74,75].

One example of regulatory elements of gene expression are TFs, which are master control proteins that regulate gene expression levels by binding to specific DNA sequences, thereby enhancing or repressing the transcriptional rates [76]. In *Chlamydomonas reinhardtii*, a microalga belonging to the group of chlorophytes, it has been observed that its responses to P starvation are regulated by the Myb family transcription factor Pi Starvation Response 1 (PSR1), which is involved in the upregulation of genes that codify phosphatases and Pi transporters to improve P acquisition and reallocation [77,78,79]. In diatoms, knowledge of the regulatory elements of gene expression remains scarce and poorly elucidated; however, Cruz de Carvalho et al. [32] found in *P. tricornutum* that, in an early stage of P deficiency, 50 TFs were upregulated, and that the majority of them belonged to the HSF, Myb and bZIP families. Later, Sharma et al. [80], through an in silico analysis, identified the TF PtPSR in *P. tricornutum* and, after analysis of mutant lines, they established that this TF is involved in P scavenging, phospholipid remodeling and cell growth adaptation to P limitation. According to Sharma et al. [80], PtPSR belongs to a clade of Myb TFs that are conserved in Stramenopiles (a taxonomic group where diatoms are classified). Moreover, they found that PtPSR is phylogenetically closed to two transcripts encoding Myb-like TFs of *Nannochloropsis oceanica* that were upregulated upon P limitation [81]. These results supported the essential role of PtPSR in response to P limitation in *P. tricornutum*.

Another example of a regulatory element of gene expression is long non-coding RNAs (lncRNAs), which are defined as RNA transcripts that contain more than 200 bp that lack protein-coding potential. It has been suggested that they are potent regulatory components of gene expression [82,83]. Cruz de Carvalho et al. [32], in *P. tricornutum* under P-limiting and P resupplying conditions, identified 1510 putative lincRNAs (long intergenic nonprotein coding RNAs), of which 202 were induced under P-limiting conditions; these were subsequently repressed when P was resupplied to the culture medium. These lncRNAs were analyzed to determine if they had a mimicry function to target sequences such as IPS1, which is a lncRNA identified in higher plants that interacts with miR399 (a microRNA whose target molecule is an mRNA that encodes a phosphate transporter); however, no function of mimicry could be predicted to a ‘target’ sequence for any of the lncRNAs identified in the study that respond to P [32]. Additionally, they investigated the correlation of co-expression between the lncRNA genes for responding to P and the neighboring genes (regulatory functions ‘cis’), finding that several of them correlate (positively or negatively) with lncRNAs. The authors concluded that this information will be very useful for future studies that seek to explain the role of these lncRNAs under P deficiency in diatoms. Although many studies have been suggested that lncRNAs are involved in important processes essential for the biological metabolism such as gene expression, more research should be done to gain more knowledge about the lncRNAs in diatoms under P deficiency.

## 5. Effect of P on Photosynthesis and Pigment Content in Diatoms

### 5.1. Molecular Strategies of Diatoms to Cope with the Impact of P Limitation on the Photosynthetic Apparatus

**Figure 1:**
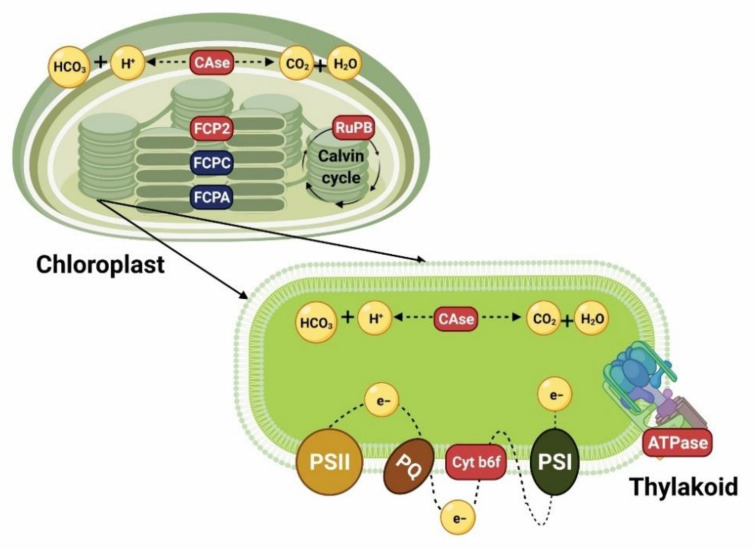
Cell model summarizing the results of transcriptomic analyses in different diatom species exposed to P limitation [31,56,57,60,61], and showing the expression of genes encoding enzymes involved in photosynthesis and pigment synthesis. Genes in red are upregulated and genes in blue are downregulated. CAse: carbonic anhydrase. FCP2: fucoxanthin–chlorophyll a–c binding protein. FCPC: fucoxanthin–chlorophyll a–c binding protein C. FCPA: fucoxanthin–chlorophyll a–c binding protein A. RuPB: ribulose-1, 5-biphosphate carboxylase. Cyt b6f: cytochrome b6-f complex iron–sulfur subunit. ATPase: ATP synthase. PQ: plastiquinone. PSI: photosystem I. PSII: photosystem II. Created with Biorender (https://biorender.com/, accessed on 26 April 2021).

P is involved in each step of photosynthesis, including as a substrate and regulator participating in processes such as light absorption, energy formation and assimilation, biochemical reactions of the Calvin cycle, synthesis of transporters and regulation of key enzymes. An immediate effect of P limitation is the decrease of the synthesis of substrates required for the Calvin cycle, and consequently a reduction in photosynthetic phosphorylation levels [84,85]. Moreover, changes to the photosynthetic rate and the synthesis of molecules such as NADP and NADPH, affecting electron transport through the photosystem II (PSII), are further effects of P limitation [84,85,86]. Figure 1 shows a summary of the regulation of some gene expression encoding enzymes involved in photosynthesis under P limitation.

A gene that has been found to be upregulated under P limitation encodes ribulose-1, 5-biphosphate carboxylase (RuPB), suggesting that under this condition carbon assimilation still occurs, and could even increase. In *P. tricornutum*, it has been reported that after 48 h of P deficiency, algal cells can divide for at least two more generations. Moreover, Zhang et al. [60] found in *S. costatum* that after resupplying P to the culture media, this gene was downregulated. Another gene that supports the abovementioned is one that encodes carbonic anhydrase (CAse), which catalyzes the reversible conversion of CO_2_ and water to bicarbonate and protons. This gene was found upregulated in *P. tricornutum* under P deficiency, suggesting that carbon fixation still continues, even though photosynthetic efficiency starts to decrease within 48 h, which was the period of time that the experiment lasted.

Other genes that are differentially expressed in diatoms cultured under P limitation involved in photosynthesis are those related to pigment binding proteins and photochemical efficiency. In *Chaetoceros affinis* and *S. costatum,* several putative genes encoding fucoxanthin chlorophyll a/c binding proteins (FCP2) were upregulated [60,61]. Additionally, Zhang et al. [60] reported the upregulation of genes which encode for P700 and P680, which are key pigment proteins of photosystems I (PSI) and II (PSII) in eukaryotic cells. Shih et al. [61] suggested that even though the maximum quantum yield (Fv/Fm) values calculated for *C. affinis* in their study indicated that electron transport may be partially blocked under P limitation, the upregulation of pigment genes is a compensatory response to the possible damage that the photosynthetic apparatus may undergo and to cope with the excess of energy that the cells will still receive. Nevertheless, in two studies of *P. tricornutum* cultured under P deficiency, these genes were found to be downregulated (FCPA, FCPC) [56,57].

Regarding electron transport in photosynthesis, among the genes that were found to be upregulated in *C. affinis* and *S. costatum* were those that encode for cytochrome b6-f complex iron–sulfur subunit (Cyt b6f) and F-type H+-transporting ATPase [60,61]. The former is involved in the regulation of electron transport between PSI and PSII, and the latter is also known as ATP synthase (ATPase) and catalyzes the hydrolysis or synthesis of ATP coupling with H^+^ transport across the membrane [87,88]. These results suggested that even though these genes are upregulated, algal cells are in a process of high-speed energy consumption and that, under severe P limitation, they enhance their chloroplast heat dissipation pathways to reduce the potential damage caused by the excess of energy [31]. Conversely, these genes and others related to electron transport in two studies of *P. tricornutum* cultured under P deficiency were found to be downregulated [56,57].

Interestingly, another study in *P. tricornutum* reported similar results in terms of gene expression related to carbon fixation, pigment binding proteins and electron transfer that have already been reported for *C. affinis* and *S. costatum* [31,60,61]. These differences when compared with other studies on *P. tricornutum* could be explained in part by the experimental design, laboratory conditions, handling and acclimatization of the strains and the selection of growth phase or time that the diatoms were exposed to P deficiency and then harvested for analysis [31,56,57].

### 5.2. Effect of P Limitation on the Photosynthetic Apparatus and Pigment Content in Diatoms

Fv/Fm is a common parameter used to assess the physiological status of the photosynthetic apparatus, and it has been used in diatoms cultured under P limitation to determine if the concentrations tested in each species and bioassay have an effect on photosynthesis. It has been established that values between 0.6 and 0.7 indicate that phytoplankton are not under nutrient-limiting conditions [89,90]. Reports in *P. tricornutum* and *T. weissflogii* have shown that at low P concentrations, the Fv/Fm ratios decreased rapidly, while at P-replete concentrations, the Fv/Fm values remained high (0.5–0.7), suggesting that the algal cells were healthy [38,58]. This same pattern was observed in *C. affinis* cultured under P limitation. Shih et al. [61] observed that under favorable P concentrations, Fv/Fm values were higher than 0.5; however, under P-limiting concentrations, the Fv/Fm values decreased considerably. They concluded that the low values of Fv/Fm could indicate a partial blockage of the electron transport chain, and that the upregulation of genes encoding components of the light-harvesting complex (LCH) could represent a compensatory response to reduce the electron flow and achieve dissipation of excess energy in order to protect the photosynthetic apparatus [61,91].

On the other hand, an interesting field in the study of marine algae is the production of pigments, which also play a leading role in photosynthesis. Pigments have been shown to possess biological activity and potential health benefits; they are therefore of great importance in many sectors of industry, such as food, cosmetics and pharmacology [92,93] (Table 2).

The main pigments of diatoms are chlorophyll *a*, fucoxanthin, diadinoxanthin, β-carotene and alloxanthin, and it has been widely reported that the content of these pigments is influenced by the P concentration [100]. Finenko and Krupatkina-Akinina [29] observed in seven diatom species that the contents of chlorophyll *a* and *c* are defined by P availability, finding higher values as the concentration of this nutrient increased. On the other hand, studies by Guerrini et al. [101] found that the chlorophyll *a* content in *Achnanthes brevipes* was more affected by N limitation than P limitation. They found no significant effect of the P concentrations evaluated. In the haptophyte *Isochrysis galbana*, chlorophyll content decreased; however, an increase in carotenoids was observed, suggesting that this may be a protection mechanism against photo-oxidative stress [102]. Recent studies in *C. muelleri* cultured in F media reported that the highest content of chlorophyll *a* was found at 72 µM Pi, while at 7 and 18 µM Pi (considered as low P concentrations in the study), the chlorophyll *a* content was significantly reduced [35].

Studies in *T. weissflogii* found that at 0.07 µM Pi and P deprivation, the chlorophyll *a* content decreased rapidly, while at 36 µM Pi the highest chlorophyll *a* content was found. Moreover, the fucoxanthin/chlorophyll *a* and diadinoxanthin/chlorophyll *a* ratios were determined, finding at low P concentrations that their values decreased rapidly, while at higher P concentrations they remained stable. Conversely, the β-carotene/chlorophyll *a* ratio showed an increase at low P concentrations. These results suggested that even though these pigments do not have P in their composition, their content depends on its availability, especially fucoxanthin and diadinoxanthin [38].

## 6. Storage Compound Metabolism in Diatoms under P Limitation

### 6.1. Effect of P on Carbohydrate Metabolism in Diatoms

#### 6.1.1. Effect of P on the Expression of Genes Involved in Carbohydrate Metabolism

The polysaccharide biosynthesis pathway in diatoms remains unknown, including the enzymes involved in this process and the effects arising from P-limited conditions in terms of polysaccharide constitution. However, the already known effects, such as the release of accumulated carbon and protection to the cell, have been well-documented [19]. Brembu et al. [19] pointed out that the genomes sequenced so far all differ in the composition of the predicted glycolysis pathway, and that various glycolytic enzymes and some variable numbers of isoenzymes are unique in one or two species, making it a very interesting field of study, since the effects of P limitation in each species could be different. Currently, the mechanism of carbon fixation in diatoms remains unknown; however, different characteristic enzymes of the C3 and C4 photosynthetic metabolic pathways have been detected among different genera and within species [103].

Among the key enzymes present in the C4 photosynthetic pathway, pyruvate orthophosphate dikinase (PPDK) and phosphoenolpyruvate carboxylase (PEPC) are the most important [19,104]. PPDK catalyzes the reversible reaction of phosphoenolpyruvate synthesis, starting gluconeogenesis from pyruvate. PEPC converts CO_2_ to phosphoenolpyruvate (PEP) and, later, PEP is converted to oxaloacetate, which is used in the carbon fixation process [19,31,105]. Yang et al. [31] found, in *P. tricornutum* cultured under P starvation, a 17.7-fold upregulation of *PEPC* compared to the control treatment; later, Alipanah et al. [56] corroborated the upregulation of this gene in another study on *P. tricornutum,* also cultured under P deficiency. Additionally, they found upregulation of a gene that codifies triose-phosphate isomerase (TPI), an important enzyme involved in energy production; this enzyme is responsible for catalyzing the conversion of glyceraldehyde-3P (G3P) into glycerone-P, which is a substrate for sucrose and chrysolaminarin production. However, in *P. tricornutum* and *T. pseudonana* cultured under P deficiency, a higher upregulation of genes involved in the degradation of chrysolaminarin compared to its biosynthesis has been observed. These results suggested that the breakdown of this compound could be a strategy for the synthesis of more complex polysaccharides [1,32].

Other genes found to be upregulated under P starvation in *P. tricornutum* codify key enzymes involved in carbohydrate metabolism, such as phosphoglucomutase (PGM), beta-glucosidase and UDP-glucose-6-dehydrogenase (UGDH), pyruvate kinase (PK), pyruvate dehydrogenase complex (PDC) and 6-phosphofructokinase (6-PFK) [31]. PFK is one of the most important enzymes involved in the glycolysis pathway, which catalyzes the irreversible conversion of fructose 6-phosphate (F6P) to fructose 1,6-biphosphate (FBP1); this enzyme is regulated by ATP/AMP ratios, fructose 2,6-biphosphate and PEP concentrations. When there is a P limitation, ATP/AMP ratios are low, triggering enzyme activation; further, it has been reported that high concentrations of fructose 2,6-biphosphate could induce PFK activity. On the contrary, when there are higher PEP concentrations, the PFK activity is inhibited [106].

Zhang et al. [60] found, in *S. costatum* cultured in P deficient medium, that genes which encode for TPI, phosphoglycerate kinase (PGK) and phosphofructokinase-1 were upregulated. Other genes that were upregulated under these conditions included fructose 1-6 bisphosphate I, fructose-biphosphate aldolase, PK and pyruvate dehydrogenase (PDH) and aldose 1-epimerase (GalM). Shih et al. [61] found that, in *C. affinis* cultured under P limitation, the expression of genes involved in the Calvin cycle was not significantly affected; however, the carbon fixation might have been negatively impacted. Moreover, they found, under low P concentrations, upregulation of 1,3-beta-glucan synthase, which it is involved in polysaccharide synthesis. It seems that upregulation of genes involved in glycolysis or gluconeogenesis is favored in order to produce energy in diatoms as a strategy to cope with P deficiency [19,56].

#### 6.1.2. Carbohydrate Accumulation under P Limitation in Diatoms

Microalgae are responsible for the large quantities of mucilage released into the oceans, with benthic diatoms acting as the main producers [21,24,107]. These exudates are composed of a variety of EPSs [19,108], including polysaccharides [21,107]. Diatoms possess a high content of polysaccharides, and chrysolaminarin (β-1,3-glucan) is their main storage carbohydrate [19,101,109,110,111,112,113]. This polysaccharide is synthesized from uridine diphosphate glucose (UDP Glc) via the gluconeogenesis pathway [19,109]. Benthic diatoms can use EPSs as a mechanism of locomotion through their expulsion from the raphe (central fissure of the diatom structure) [24,108]. This mucilage can help diatoms to bind to sediment and can protect them from stressful environmental conditions [19,107,114].

EPSs are composed of a mixture of monosaccharides, forming heteropolysaccharides with a high degree of branching [19]; however, their composition is highly variable, depending on the species, culture age and environmental conditions [21,101]. The monosaccharide composition can vary between diatom species, but the major constituents are generally glucose, galactose, fucose and mannose [19,101,107,108,110]. It is known that diatoms possess enzymes involved in the biosynthesis of monosaccharides that constitute extracellular polysaccharides [110]; their production is carried out through gluconeogenesis [19] or by chrysolaminarin degradation [108].

It has been observed that nutrient limitation induces the production and accumulation of polysaccharides in many species of diatoms; however, few investigations have been carried out on P limitation in diatoms and its relationship with polysaccharide accumulation [19,21,101]. Urbani et al. [21] observed that under P-limited culture conditions, the diatoms *T. pseudonana*, *S. costatum* and *C. closterium* produced a large amount of polysaccharides compared with a nutrient-replete culture (F/2 from Guillard and Ryther). Their results confirm that the P concentration is a determining factor in the synthesis of polysaccharides. On the other hand, Guerrini et al. [101] and Guerrini et al. [23] found that both the diatoms *Cylindrotheca fusiformis* and *A. brevipes* release significant amounts of extracellular polysaccharides under P-limited conditions. These studies have shown that the activity of the enzymes involved in the metabolic pathway of glycolysis is reduced as a consequence of a metabolic switch from protein to carbohydrate production produced by low P concentrations [101].

**Figure 2:**
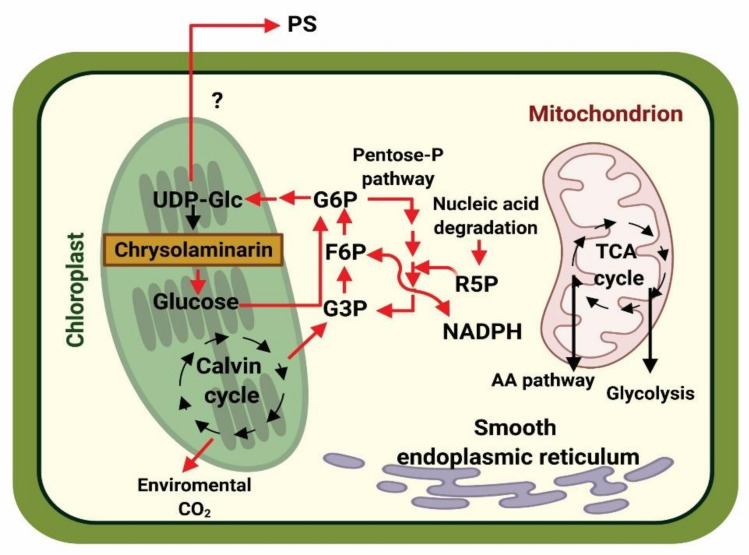
Hypothetical model of the changes in carbon flux of diatoms in P-limiting conditions. The red arrows indicate the main carbon flow. The (?) symbol indicates an unknown pathway. PS: polysaccharides. UDP-Glc: uridine diphosphate glucose. G6P: glucose-6-phosphate. F6P: fructose-6-phosphate. G3P: glyceraldehyde-3-phosphate. R5P: ribose-5-phosphate. TCA: tricarboxylic acid cycle. NADPH: nicotinamide adenine dinucleotide phosphate. This image was designed by referencing the report of Brembu [19]. Created with Biorender (https://biorender.com/, accessed on 26 April 2021).

Figure 2 shows a hypothetical model of the changes in carbon flux during P limitation in diatoms. Due to augmented levels of citrate in the Krebs cycle, inhibition of PFK is induced, and this promotes the switch from the cytosolic glycolysis pathway to gluconeogenesis. During this process, glucose-6-phosphate (G6P) may enter the oxidative pentose phosphate pathway (OPPP), which is supplemented with ribose 5-phosphate (R5P, produced from nucleic acid degradation) [19]. Glucose-6-phosphate dehydrogenase (G6P) can be converted into uridine diphosphate glucose (UDP-Glc), which is a substrate for the biosynthesis of chrysolaminarin, as well as the sugars found in polysaccharides. These biomolecules are later synthesized and finally secreted into the extracellular medium. Another interesting aspect of EPSs release is that the polysaccharides produced by diatoms could promote a symbiotic relationship with heterotrophic bacteria, wherein these microorganisms could use the released carbohydrates as a carbon source. As bacteria consume carbohydrates, inorganic compounds such as phosphate are produced, which can be used by microalgae exposed to nutrient stress [19,24,101]. In this way, the symbiotic relationship ensures quick recycling of nutrients and their release into the environment [101].

### 6.2. Effect of P on Lipid Metabolism in Diatoms

#### 6.2.1. Transcriptional Changes in Lipid Metabolism Influenced by P Availability

**Figure 3:**
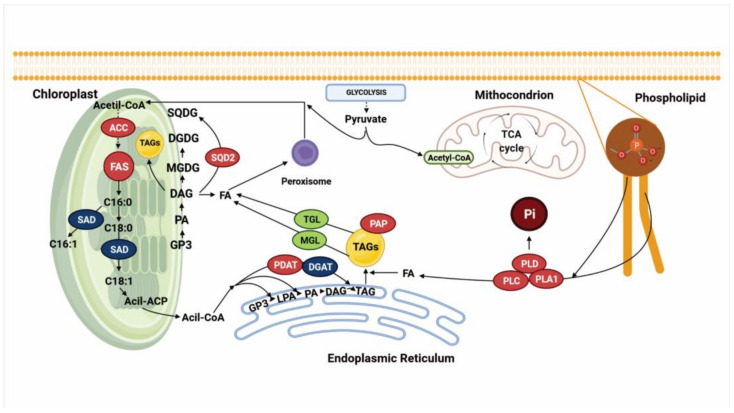
Cell model summarizing the results of transcriptomic analyses in different diatom species exposed to P limitation [1,31,56,57,60,61] and showing the expression of genes encoding enzymes involved in lipid metabolism. Genes in red are upregulated, genes in blue are downregulated, and genes in green did not show a change in their expression. ACC: acetyl-CoA carboxylase. FAS: fatty acid synthase complex. SAD: stearoil-ACP desaturase. SQDG: sulfolipid sulfoquinovisildiacylglycerol. DGDG: digalactosyldiacylglycerol. MGDG: monogalactosyl-diacylglycerol. DAG: diacylglycerol. PA: phosphatidic acid. GP3: glyceraldehyde-3-phosphate. SQD2: sulfoquinovosyl transferase 2. FA: fatty acids. PDAT: phospholipase:acylglycerol acyltransferase. DGAT2: diacylglycerol-O-transferase 2. PAP: PAP fibrillin. MGL: monoacylglycerol lipase. TGL: TAG lipase. PLD: phospholipase D. PLC: phospholipase C. PLA1: phospholipase A1. TAGs: triacylglycerides. Created with Biorender (https://biorender.com/, accessed on 26 April 2021).

Although the responses to P limitation in diatoms have been found to be quite similar among the species studied so far, several transcriptomic studies have shown that the expression of key genes involved in lipid metabolism can be dependent on the species, P concentrations, the culture conditions and the duration of the experiment [1,56,60]. Figure 3 shows a summary of the results of several transcriptomic studies in diatoms under P limitation, and the expression of genes involved in lipid metabolism.

Across several studies on *P. tricornutum* cultured under P limitation, 11 putative phospholipases were found, and their expression varied among the different studies. Yang et al. [31] found that all these phospholipases were upregulated, and they identified that these enzymes are involved in phospholipid degradation, providing fatty acids to synthesize TAGs and the release of Pi for cell growth. Later, Feng et al. [57] corroborated this response in the same species and, additionally, they found that annexin, a key enzyme involved in modulation of the activity of some phospholipases, such phospholipase A2, was downregulated, supporting the fact that phospholipid degradation may be occurring under this condition [57,115,116]. Later, Alipanah et al. [56] found that the expression of these 11 putative phospholipases varied; 6 were upregulated, 3 remained unchanged, and 2 were downregulated. Of those that were upregulated, most encoded phospholipases C (PLC) and D (PLD). Other studies have also reported their upregulation as an early response to P limitation, promoting the degradation of phospholipids to release diacylglycerol (DAG), phosphatidic acid (PA) and fatty acids, in order to facilitate TAG accumulation [19].

In *T. pseudonana*, a putative protein encoded by the *sqdB* gene was identified, which was induced under P-limiting conditions and is related to the biosynthesis of sulfolipids [1]. Conversely, in *C. affinis,* the *SQD2* gene did not show a change in its expression. In *S. costatum,* genes that encode proteins involved in the sulfolipid biosynthesis such as sulfoquinovosyltransferase, UDP-sulfoquinovose, UDP-sulfoquinovose synthase and desulfoglucosinolate were significantly upregulated, and genes involved in betaine biosynthesis were identified and showed high expression under P limiting conditions [60]. Alipanah et al. [56] found two isoforms of the *SQD* gene in *P. tricornutum* (*SQD1* and *SQD2*), and they reported that under P limitation, the expression of *SQD1* did not change in their transcript’s levels, but *SQD2* was upregulated.

Regarding the effect of P limitation on fatty acid synthesis, several studies have reported an increase in the total content; however, the amount of transcripts involved in fatty acid biosynthesis remains unchanged or decreases [1,56,61]. Some studies suggest that the repression of this metabolic pathway may be due to the arrest of cell division, decreased fatty acid requirements and experimental conditions and may depend on the growth phase in which the sample is harvested [19,61]. One of the crucial enzymes involved in fatty acid synthesis is acetyl-CoA carboxylase (ACC), which catalyzes the formation of malonyl-CoA from acetyl-CoA. It has been shown that ACC is an important enzyme that regulates the carbon flux into fatty acid biosynthesis, and that its activity is related to the amount of fatty acids synthesized [117]. Yang et al. [31] cultured *P. tricornutum* under P deficiency, finding two isoforms of *ACC* were upregulated. These results suggested that the increase of TAGs can be partially attributed to the de novo biosynthesis of fatty acids.

Among the important genes involved in fatty acid synthesis that have been identified in transcriptomic studies of diatoms grown under P limitation is ketoacyl-ACP synthase (KAS), a subunit of the fatty acid synthase complex (FAS), which catalyzes the first and rate-limiting step in fatty acid elongation to form ketobutyryl-ACP from malonyl-ACP; later, this compound is converted through a series of reactions that are repeated several times until palmitoyl-ACP is formed [117,118,119]. In *P. tricornutum* cultured under P deficiency, *KAS* is upregulated [31]. Another gene that was found in studies by Yang et al. [31] was *SAD*, which encodes stearoyl-ACP desaturase; this enzyme is involved in the introduction of the first double bond into C18:0-ACP to form C18:1-ACP [117,120]. In *P. tricornutum*, *SAD* was found to be downregulated, suggesting that this response is related to the accumulation of saturated fatty acids, mainly palmitic acid [31].

On the other hand, the key enzymes involved specifically in TAG synthesis include phospholipase:acylglycerol acyltransferase (PDAT), diacylglycerol-O-transferase 2 (DGAT2) and PAP fibrillin. PDAT catalyzes the transfer of a fatty acyl moiety from the sn-2 position of phosphatidylcholine (PC) to the sn-3 position of sn-1 of DAG, to form TAGs [121]. In *C. affinis* cultured under P limitation, the gene that encodes PDAT does not show a change in its expression [61]; however, Alipanah et al. [56] reported an increase in its expression in *P. tricornutum*. DGAT2 is an enzyme that catalyzes the last step in TAG biosynthesis, and it has been of particular interest in several organisms as it promotes TAG accumulation, mainly in plants and microalgae [122]. Under P limitation in *C. affinis*, two isoforms of DGAT2 were downregulated; the authors suggested that if TAG accumulation is occurring in *C. affinis* under P limitation, it may be due to a reduction in lipid consumption rather than an improvement in fatty acid biosynthesis [61]. It is well documented that PDAT and DGAT are crucial enzymes in TAG synthesis, not only in microalgae but also in higher plants and yeasts [117,121,122,123]. The contribution of both enzymes in TAG synthesis, even in plants, is still a matter of debate, since different isoforms have been found among species [56,61,117,123,124]. Further, due to the importance of PDAT and DGAT and their respective isoforms identified by transcriptomics analyses, genetic engineering studies have been developed to promote lipid accumulation in both plants and microalgae [125,126,127,128].

Finally, another interesting gene that encodes a putative PAP fibrillin was found to be upregulated under P limitation in *P. tricornutum* [57]; this enzyme is responsible for promoting stability of the lipid droplets (LDs) (which are organelles that stores neutral lipids) inside the algal cells, and this result was corroborated by another study investigating the same species, which found that under P limitation, the size and the number of LDs increased [31,129,130].

#### 6.2.2. Influence of P Availability on Lipid Accumulation in Diatoms

It has been widely reported that among the responses found in phytoplankton under P limitation or deficiency are cell membrane remodeling and lipid accumulation; the first occurs with the degradation of phospholipids and their substitution by non-P lipids, while the second involves the storage of lipids in the form of TAGs, representing a source of energy in adverse conditions. Under P-deficient conditions, microalgae cells are known to accumulate TAGs, and the proportion of phospholipids is reduced [13]. In diatoms, the accumulation of neutral lipids represents from 12 to 59% of total lipids, and in some species, it represents up to 81%. This feature has generated great interest in the energy sector, since their potential as an alternative energy source appears promising [131,132,133,134].

Lipid catabolism is a process that occurs in all organisms, through which cells break down TAGs and other lipid sources, such as membrane lipids, to provide substrates such as free fatty acids and Pi for themselves in order to maintain various biological processes necessary for their proper functioning and survival [132,135]. Phospholipids represent a significant amount of cellular P that is mobilized in response to low concentrations of this nutrient, and its degradation appears to release a significant amount of P to allow the microalgal cells to divide even under poor conditions [135,136]. A distinctive feature of phytoplankton in response to low P concentrations is the replacement of membrane lipids by those that do not possess P in their composition. Within the composition of phospholipids in eukaryotic phytoplankton, those found in greatest abundance are phosphatidylcholine (PC), phosphatidylglycerol (PG) and phosphatidylethanolamine (PE) [135,137]. Under P limiting conditions, *T. pseudonana* replaces PC with betaine lipids such as diacylglycerolcarboxyhydroxymethylcholine (DGCC), and PG by sulfolipid sulfoquinovisildiacylglycerol (SQDG). Additionally, it has been observed that *T. pseudonana* responds quickly to P resupply to the culture medium, degrading DGCC and facilitating the synthesis of phospholipids [137].

Concerning the fatty acid composition of diatoms, the high content of fatty acids of 16 carbon atoms is a distinctive feature of this species, with the most abundant being palmitoleic and palmitic acids. Conversely, the content of fatty acids of 18 carbon atoms is in low or trace quantities in these species. Other fatty acids found in diatoms in smaller amounts, compared to palmitoleic and palmitic acids, are myristic, arachidonic and docosahexaenoic (DHA) acids. Another characteristic fatty acid is eicosapentaenoic acid (EPA), which can represent more than 10% of the total fatty acid profile of diatoms [132,138,139]. It has been widely reported in microalgae that culture phases and different abiotic factors, such as temperature, light, CO_2_ supply and nutrient availability (such as P), influence the fatty acid content, and diatoms are not an exception [34,140,141,142].

P limitation leads to inhibition of synthesis of n-3 polyunsaturated fatty acids (PUFA) [143]. Siron et al. [144], in *P. tricornutum* cultured in an enriched media [145], found that under P deprivation, the content of palmitoleic and palmitic acid increased, while the content of EPA decreased drastically, compared to culturing in P replete media. The authors suggested that the increase in the content of palmitoleic and palmitic acids in *P. tricornutum* is related to TAG accumulation. Moreover, these authors stated that P deprivation drastically affects EPA levels, observing that under this condition levels of this fatty acid were reduced by half compared with the control treatment. Later, Cañavate et al. [146] corroborated this same pattern in *P. tricornutum* by culturing this diatom in F/2 medium under P deprivation, observing that the content of palmitoleic acid doubled and the content of EPA was reduced almost by half compared to the control treatment (100 µM P). Conversely, in *Chaetoceros gracilis* it was observed that the content of palmitic acid increased considerably compared to the content of palmitoleic acid under P deprivation, and as in other studies carried out on diatoms, the content of EPA was reduced. Lin et al. [140] carried out a study of the effect of P deprivation on *C. muelleri* and *T. weissflogii,* and its effect on their fatty acid profiles. They observed in *C. muelleri* a similar response to that already reported in *C. gracilis*; however, in *T. weissflogii*, the content of palmitoleic acid increased considerably, unlike that of palmitic acid in the absence of P in the culture medium.

On the other hand, the effect of P excess on microalgae and its fatty acid profile has been little explored. Lopes et al. [34] evaluated three P concentrations (76, 114 and 228 µM) and two growth phases (exponential and stationary) and their effect on the fatty acid profile in *P. tricornutum* cultured in F/2 medium. The results obtained in this study showed that even though the P concentration increased from 1.5-fold to 3-fold higher than the control treatment, no significant differences were detected in the content of fatty acids between the different P concentrations. However, significant differences were found when contrasting the exponential phase with the stationary phase, with the latter showing a decrease in the content of EPA and an increase in palmitic and palmitoleic fatty acids. These results corroborate that another important factor in fatty acid content is the growth phase during which the sample is harvested, as this will be related to the concentration of nutrients in the culture medium.

## 7. Conclusions

P is an essential nutrient in cell growth and metabolic processes such as energy transfer, macromolecules biosynthesis and photosynthesis in microalgae. It has been reported that P limitation in diatoms is an efficient strategy to induce the synthesis of biomolecules and the accumulation of storage compounds, such as pigments, carbohydrates and lipids, with diverse biological activities, which can be used in several biotechnological applications. However, one of the challenges to overcome in this process is the effect on biomass production of limitation of nutrients. Obtaining an adequate yield of by-products from the cultivation system of the diatoms could be another challenge. On the other hand, the information provided by omics sciences and the integration of the results obtained in relevant research have led to a broader understanding of the mechanisms by which diatoms cope with P limitation, since each species may demonstrate different responses. Finally, it should be noted that, in the future, it will be of interest to carry out studies that utilize genetic engineering, in order to carry out genetic improvement of the strains. We suggest more genetic engineering studies on lipid metabolism in diatoms (considering *ACC*, *PDAT* and *DGAT* genes) to improve lipid accumulation, since it has been widely reported that commercial products such as biofuels (biodiesel), food supplements and pharmaceuticals (EPA and DHA) can be obtained from diatoms.

## Figures and Tables

**Table 1:** Genes involved in P assimilation and cellular homeostasis, reported in diatoms.

Gene Name	Protein Name	Function	Diatoms	References
*Npt2b*	Sodium-dependent phosphate transporter	Na and Pi transport	*T. pseudonana* *P. tricornutum* *C. affinis*	[1,31,32,61]
*PHT1*	Inorganic phosphate transporter	Pi transport	*S. costatum*	[60]
*SLC25A3*	Solute carrier family 25, member 3	Pi transport	*S. costatum*	[60]
*APa*, *PtAPase*, *scoap*, *THAPSDRAFT_261067*, *PHATDRAFT_49678*	Alkaline phosphatase	DOP hydrolysis	*T. pseudonana* *P. tricornutum* *C. affinis* *S. costatum*	[1,31,32,56,58,60,61]
*THAPS_38194*	5′-Nucleotidase	DOP hydrolysis		[1]
*PHATR_44174*	5′-Nucleotidase	DOP hydrolysis	*P. tricornutum*	[56]
*VTC4*	Vacuolar transporter chaperone 4	Pi storage and cellular homeostasis	*T. pseudonana* *P. tricornutum*	[1,32]
*VPT1, PHT5*	Vacuolar phosphate transporter	Pi transport into the vacuole	*S. costatum*/ *P. tricornutum*	[56,60]

**Table 2:** Activity of some pigments present in diatoms.

Pigments	Source	Biological Activity	Effect of P Limitation	Reference
Fucoxanthin	*Odontella aurita*	Antioxidant Antiproliferative	Low content	[94,95]
	*Chaetoceros calcitrans*			[96]
Marennine	*Haslea karadagensis*	Antiviral Antibacterial Antifungal	N.A.	[97]
	*Haslea ostrearia*	Antioxidant		[98]
Chlorophyll derivative (Pheophorbide *a*)	*C. closterium*	Anti-inflammatory	N.A.	[99]

N.A: Not Available.

## Data Availability

Not applicable.
